# A generic RNA-pulsed dendritic cell vaccine strategy for renal cell carcinoma

**DOI:** 10.1186/1479-5876-3-29

**Published:** 2005-07-26

**Authors:** Christiane Geiger, Sybille Regn, Andreas Weinzierl, Elfriede Noessner, Dolores J Schendel

**Affiliations:** 1 Institute of Molecular Immunology, GSF-National Research Center for Environment and Health, Munich, Germany; 2 Institute of Cell Biology, Department of Immunology, University of Tübingen, Tübingen, Germany

**Keywords:** dendritic cells, tumor-derived RNA, renal cell carcinoma, tumor vaccine, immunotherapy

## Abstract

We present a generic dendritic cell (DC) vaccine strategy for patients with renal cell carcinoma (RCC) based on the use of RNA as a source of multiplex tumor-associated antigens (TAAs). Instead of preparing RNA from tumor tissue of each individual RCC patient, we propose to substitute RNA prepared from a well characterized highly immunogenic RCC cell line (RCC-26 tumor cells) as a generic source of TAAs for loading of DCs. We demonstrate here that efficient RNA transfer can be achieved using lipofection of immature DCs, which are subsequently matured with a cytokine cocktail to express high levels of MHC and costimulatory molecules as well as the chemokine receptor CCR7. Neither RNA itself nor the lipid component impacted on the phenotype or the cytokine secretion of mature DCs.

Following RNA loading, DCs derived from HLA-A2-positive donors were able to activate effector-memory cytotoxic T lymphocytes (CTLs) specific for a TAA ligand expressed by the RCC-26 cell line. CTL responses to RNA-loaded DCs reached levels comparable to those stimulated directly by the RCC-26 tumor cells. Furthermore, DCs expressing tumor cell RNA primed naïve T cells, yielding T cell lines with cytotoxicity and cytokine secretion after contact with RCC tumor cells. RCC-26 cell lines are available as good manufacturing practice (GMP)-certified reagents enabling this source of RNA to be easily standardized and adapted for clinical testing. In addition, well defined immune monitoring tools, including the use of RNA expressing B cell lines, are available. Thus, this DC vaccine strategy can be directly compared with an ongoing gene therapy trial using genetically-engineered variants of the RCC-26 cell line as vaccines for RCC patients with metastatic disease.

## Background

Renal cell carcinomas (RCC) are classified as immunogenic tumors based on the observation that patients with metastatic RCC show some of the most favorable responses to immunotherapy [reviewed in [1,2]]; tumor-infiltrating lymphocytes (TIL) have been isolated that kill autologous tumor cells following restimulation *in vitro *[reviewed in [3]] and adoptive transfer of TIL provided clinical benefit to some RCC patients [4-7]. It would be desirable to exploit these reservoirs of effector cells in RCC patients in order to improve antitumor immunity. This could be achieved either by vaccinating patients to boost pre-existing cellular immunity or by using adoptive transfer of specific effector cells that have been activated and expanded *ex vivo*.

Applying specific antigens for these purposes has been hindered in RCC by a paucity of information regarding the identity of tumor-associated antigens (TAAs) that can serve as effective tumor rejection antigens. For example, members of the cancer-germline family, like NY-ESO-1 molecules and several members of the MAGE family which display suitable characteristics as immunogens for other tumors, are not expressed in the majority of RCC [8-12]. Therefore, vaccine developments for RCC have concentrated to date on the use of tumor cells themselves to provide mixtures of unknown TAAs as immunogens. On this basis, several types of autologous RCC vaccination strategies have been evaluated in clinical trials, including the use of inactivated tumor cells, gene-modified tumor cells or DCs expressing antigens derived from RCC lysates, tumor-RNA or following fusion with autologous tumor cells [reviewed in [13]]. While these approaches were found to be feasible, demonstrated limited toxicity, and showed some bioactivity and clinical impact, long-term vaccination was hindered by the lack of adequate amounts of tumor from many patients. To overcome this limitation, alternative strategies using allogeneic tumor cell lines can be employed, whereby development of effective antitumor immunity relies on the presence of target molecules that are shared among various RCC. CTL recognition studies and molecular profiling of tumor cells support the validity of this contention for RCC [14-22].

We developed an allogeneic tumor cell vaccine using a well characterized tumor line (RCC-26) that was derived from a patient with early stage disease (T1, N0, M0). Studies of autologous and allogeneic TIL demonstrated that this RCC line displayed a number of distinct peptide-major histocompatibility complex (pMHC) ligands, some of which were restricted by HLA-A*0201-encoded molecules and shared by other RCCs. Autologous TIL-26 cells recognized their specific pMHC ligands on RCC-26 cells but not on cells derived from normal kidney parenchyma (NKC-26) or Epstein- Barr virus-transformed lymphoblastoid cells (LCL-26) [14], revealing their tumor-associated specificity. We genetically engineered RCC-26 cells to express CD80 together with selected cytokines in order to enhance their natural immunogenicity and we have initiated a two-center trial comparing vaccine variants expressing CD80 with IL-2 or IL-7 in HLA-A*0201-matched patients with metastatic disease [13,23].

While this allogeneic approach has the advantage that vaccines can be derived from well charcterized tumor cells and a generic source of vaccine can be used in multiple patients, it is limited by the fact that immunization may only be optimally effective in patients who are partially matched for HLA allotypes with the vaccine cells, since encounter with DCs allowing cross-presentation of TAAs may not be very efficient *in vivo*. Furthermore, if the vaccine cell is the direct APC for T cell stimulation, responses are limited to CD8^+ ^T cells because the RCC cells do not express MHC class II molecules.

To overcome this limitation and allow patients with various HLA allotypes to be entered into clinical trials, the development of a vaccine using autologous DCs loaded with tumor-derived RNA represents a promising new approach [24]. We reasoned that RNA derived from our well characterized RCC-26 cell line could be used not only as a source of multiplex TAAs, but also could serve as a tool for post-vaccination immune monitoring. Here we report our strategy for the development of a generic RNA-pulsed DC vaccine and the characterization of DCs loaded with RCC-derived RNA.

## Methods

### Cell lines and cultures

The renal cell carcinoma cell line RCC-26 was established from a primary stage I (T1, N0, M0, G2) clear cell carcinoma of patient-26, as described previously [14] (HLA alleles: A*0201, A*3303, B*4101, B*5101, Cw*1502 and Cw*1701). The RCC- 53 cell line was generated similarly from a second patient (T2, N1, M1, G2-3) with clear cell carcinoma (HLA alleles: A*0201, A*2501, B*1501, B*1801, Cw*1203 and Cw*0303) [25]. Epstein-Barr virus (EBV)-transformed lymphoblastoid cell lines (LCLs) were generated as described [26]. All cell lines were cultured in RPMI 1640 medium supplemented with 12% fetal bovine serum (FBS), 2 mM L-glutamine, 1 mM sodium pyruvate and 1× non-essential amino acids. The TIL-26 cell line was obtained from the primary tumor of patient-26 as published [14], cultured in RPMI 1640 medium containing 7.5% FBS, 7.5% heat-inactivated, pooled human serum, 2 mM L-glutamine, 1 mM sodium-pyruvate and 1× non-essential amino acids and restimulated every 14 days with irradiated autologous tumor cells.

### Generation of monocyte-derived DCs from peripheral blood mononuclear cells

Peripheral blood mononuclear cells (PBMCs) were isolated by standard Ficoll-Paque (PAN Biotech, Aidenbach, Germany) density gradient centrifugation of heparinized blood obtained from healthy donors and washed twice in PBS. Subsequently, CD14^+ ^cells were affinity purified utilizing the magnetic cell sorting kit (MACS) (Miltenyi Biotec, Bergisch Gladbach, Germany), according to the manufacturer's instructions. In brief, the isolated PBMCs were incubated with CD14 MicroBeads in a concentration of 15 μl/10^7 ^PBMCs in PBS containing 1% FBS at 4°C for 15 min. The labelled cells were washed once, passed through a positive selection column, washed three times and eluted from the column. To generate DCs, the CD14^+ ^cells were cultured in X-Vivo 15 medium (Cambrex Bio Science, Verviers, Belgium) supplemented with 800 U/ml rhuGM-CSF (Leukine, Berlex, Richmond, USA) and 10 ng/ml rhuIL-4 (Promocell Bioscience, Heidelberg, Germany) at a cell concentration of 1.5 × 10^6^/ml in 6-well culture plates (TPP, Peske, Aindling, Germany). For maturation of immature DCs (iDCs), the cells were incubated on day 6–7 with a cytokine cocktail containing 250 U/ml rhuTNF-α, 20 ng/ml rhuIL-1β (Promocell Bioscience), 1 μM PGE2 (Sigma, Taufkirchen, Germany) and 1000 U/ml rhuIL-6 (R&D Systems, Minneapolis, USA) for 48 h [27]. Additionally, the cultures were supplemented with 800 U/ml rhuGM-CSF and 10 ng/ml rhuIL-4.

### Isolation of total tumor RNA

Total tumor RNA was extracted from the human RCC cell lines, RCC-26 and RCC- 53, using Tri Reagent^® ^(Sigma) according to the manufacturer's instructions.

### Generation of in vitro-transcribed mRNA

*In vitro*-transcribed enhanced green fluorescent protein (EGFP) mRNA was prepared using the vector pGEM4Z/GFP/A64 [28] (kindly provided by E. Gilboa, The Center for Cellular and Genetic Therapies, Duke University Medical Center, Durham, NC, USA), which already contained the poly(A) template. *In vitro *transcription under the control of a T7 promoter was carried out using the mMESSAGE mMACHINE™ T7 kit (Ambion, Austin, Texas, USA). IVT mRNA was then purified using the RNeasy Mini Kit (Qiagen, Hilden, Germany) following the RNA cleanup protocol.

### Transfection of DCs

Transfection of DCs with RNA was carried out using the cationic lipid reagent DMRIE-C (Invitrogen, Karlsruhe, Germany). On day 7, iDCs were harvested and resuspended in OptiMEM^®^I (Invitrogen) at a final concentration of 1.0 × 10^6 ^DCs/ml. This cell suspension was distributed immediately in 24-well plates (0.5 ml/well = 0.5 × 10^6 ^iDCs/well). The RNA/lipid solution was prepared as follows: for each DC culture well 0.5 ml OptiMEM^®^I was mixed with 5 μl DMRIE-C in a polystyrene tube and the desired amount of RNA was subsequently added to the mixture. In general, 2.5 – 5.0 μg of IVT mRNA or 5.0 μg of total tumor RNA were applied for transfection of 0.5 × 10^6 ^iDCs. The RNA/lipid solution was transferred immediately to the iDCs. After a 4 h incubation period at 37°C, the transfection solution was removed and replaced by X-VIVO 15, supplemented with the cytokine cocktail for DC maturation as indicated.

### Flow cytometric analysis

Phenotypic analysis of DCs was carried out by immunofluorescence staining of dendritic-monocyte differentiation and activation markers. Cells were labelled with mouse monoclonal antibodies (mabs) either unconjugated or directly conjugated with fluorescein isothiocyanate (FITC) or phycoerythrin (PE). The following FITC-conjugated mabs were used: anti-HLA-DR (IgG2a, clone G46-6), anti-CD83 (IgG1, clone HB15e), anti-DC-SIGN (IgG2b, clone DCN46), anti-CD14 (IgG2a, clone M5E2) (BD Pharmingen, San Diego, USA). The following mabs were used as PE-conjugated reagents: anti-CD86 (IgG1, clone 2331 FUN-1), anti-CD80 (IgG1, clone L307.4) (BD Pharmingen), anti-CD40 (IgG1, clone mAb89) (Beckman Coulter, Krefeld, Germany). Clone 2H4 specific for human CCR7 (BD Pharmingen) was unconjugated. Indirect immunofluorescence was performed using monoclonal biotin-conjugated rat-anti-mouse IgM (clone II/41) in combination with PE-conjugated streptavidin (BD Pharmingen). PE/FITC isotype-matched mouse immunoglobulins were used as negative controls (BD Pharmingen).

Cells were washed once with PBS containing 1% FBS and incubated with the appropriate mab for 30 min on ice in PBS/1% FBS. Then cells were washed once again and fixed in PBS/1% paraformaldehyde. Samples were acquired using a FACSCalibur instrument (BD Bioscience Immunocytometry Systems, San Jose, USA) and data analysis was performed using CellQuest Pro software. For determination of EGFP expression, DCs were harvested approximately 24 h after transfection, washed once and directly analyzed by flow cytometry.

### Analysis of cytokine expression

Release of cytokines by DCs was measured using the highly sensitive Bio-Plex human cytokine reagent kit (BioRad Laboratories Inc., Hercules, CA, USA), according to the manufacturer's protocol. Data analysis was performed using the BioRad Array Operation System (BioRad) and applied five parameter logistic regression algorithms.

### T cell stimulation assay

Stimulating cells were harvested and plated in triplicates in 96-well U-bottomed plates at a concentration of 1.5 × 10^4 ^cells per well in 100 μl of the appropriate T cell media. T cells were added in a concentration of 3000 cells/well (in the case of the TIL-26(GG) clone) or 1.5 × 10^4 ^cells/well (in the case of *de novo *primed T cell lines) in 100 μl, yielding a total volume of 200 μl/well. Culture supernatants were harvested after a 24 h incubation period at 37°C and release of interferon-gamma (IFNγ) was measured using a commercially available enzyme-linked immunosorbent assay (BD Pharmingen).

### T cell priming

The generation of antitumor T cell lines was carried out using autologous cells derived from blood of healthy volunteers. Monocyte-derived iDCs were transfected with total tumor RNA, as described above and matured for 48 h. In 24-well plates, 1.25 × 10^5 ^mDCs were incubated with 1 × 10^6 ^autologous PBMCs in a total volume of 2 ml/well in RPMI 1640 medium containing 12% heat inactivated, pooled human serum, 2 mM L-glutamine, 1 mM sodium-pyruvate and 1× non-essential amino acids. After 10–12 days of culture, cells were restimulated with autologous mDCs transfected with total cellular RNA in a ratio of 4:1 (PBMC:mDC). Subsequent rounds of restimulation were carried out in an analogous manner, replacing stimulator DCs every 7 days. 10 ng/ml rhuIL-7 (Promocell Bioscience) and 20 U/ml rhuIL-2 (Proleukine^®^, Chiron, Emeryville, CA, USA) were added to the culture three times per week starting at days 12 and 18, respectively. The cytolytic activity of induced CTLs was analyzed on day 4 after the last round of restimulation in a standard ^51^chromium-release assay. Release of IFNγ following T cell stimulation was measured on day 10 after the last round of restimulation, using a commercially available enzyme-linked immunosorbent assay (BD Pharmingen).

### ^51^Chromium-release assay

Cell-mediated lysis was measured in a standard ^51^Cr-release assay as described earlier [29]. In brief, target cells were labelled with Na_2_^51^CrO4 (Hartmann Analytic, Braunschweig, Germany) at 37°C for 1 h and co-cultured with the effector cells at the indicated effector:target (E:T) cell ratios in a 96-well V-bottomed plate for 4 h. Spontaneous ^51^Cr-release was obtained by incubating target cells alone and maximum release by directly counting labelled cells only. The percentage of specific lysis was calculated as: 100 × (experimental counts per minute (cpm) - spontaneous cpm) / (maximal cpm - spontaneous cpm). Triplicate measurements of four-step titrations of effector cells were used for the experiments.

## Results

### Strategy for a generic RNA-pulsed DC vaccine for RCC patients

**Figure 1:**
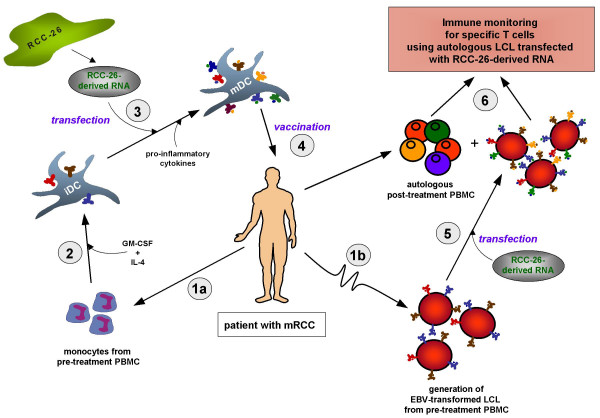
***Strategy for a generic RNA-pulsed DC vaccine for metastatic renal cell carcinoma***. See text for detailed explanation.

We foresee the following strategy for the development and application of a generic RNA-pulsed DC vaccine for RCC (Figure 1). In a first step, PBMCs from a metastatic RCC (mRCC) patient are isolated before treatment to acquire monocytes (Step 1a) and to generate autologous EBV-transformed LCLs for long-term studies (Step 1b). In a second step, the isolated monocytes are differentiated into immature dendritic cells (iDCs) in the presence of GM-CSF and IL-4 (Step 2). On day 7 of culture, iDCs are transfected with total RNA derived from the RCC-26 cell line and subsequently matured using a defined pro-inflammatory cytokine cocktail for 48 h (Step 3).

The application of RNA derived from an established and well characterized RCC cell line comprises the generic component of this approach. Total RNA is isolated from this cell line, providing a virtually unlimited source of TAAs. This bypasses the need for RNA preparation from, often limited, individual patient tumor tissues. Fully matured DCs, loaded with generic RNA can than be applied for vaccination of mRCC patients (Step 4).

The autologous LCLs are instrumental for analysing the activation, expansion and perseverance of specific T cell responses after vaccination. Transfection of autologous LCLs with RCC-derived RNA (Step 5) will generate APCs expressing different pMHC complexes with various TAA epitopes. By co-cultivating these APCs with autologous post-treatment PBMCs, under limiting dilution culture conditions, it should be possible to track the development of both MHC class I and class II-restricted T cells contributing to antitumor immunity (Step 6). Either RCC-26-derived RNA or single-species RNAs corresponding to individual TAAs expressed by RCC-26 cells [23] can be employed to potentially identify the fine specificity of emerging T cell responses.

### Immature DCs can be efficiently transfected with *in vitro *transcribed mRNA using lipofection

**Figure 2:**
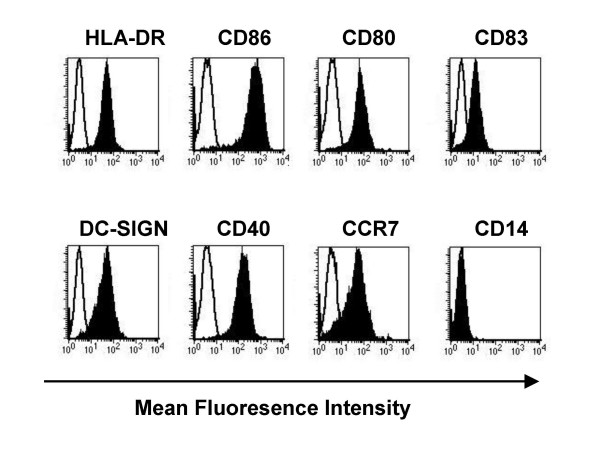
***Phenotype of mature DCs***. The incubation of monocyte-derived iDCs with a pro-inflammatory cytokine cocktail composed of IL-1β, IL-6, TNF-α and PGE_2 _for 48 h induced a fully mature DC phenotype. Status of maturation was measured by staining of different DC maturation and activation markers followed by flow cytometric analysis.

Steps 1 and 2 of the strategy described above are standard procedures nowadays. In our studies, the generation of monocyte-derived iDCs in the presence of GM-CSF and IL-4 yielded considerable cell numbers (approximately 50% of the isolated monocytes) and subsequent activation of the iDCs could be achieved by stimulation with a pro-inflammatory cytokine cocktail (TNF-α, IL-1β, PGE_2 _and IL-6) for 48 h [26]. This activation resulted in fully mature DCs (mDCs) showing high expression of maturation and activation markers, like CD86, CD80, CD83, CD40 and the chemokine receptor CCR7. In contrast, the monocyte marker CD14 was completely down-regulated (Fig. 2).

**Figure 3:**
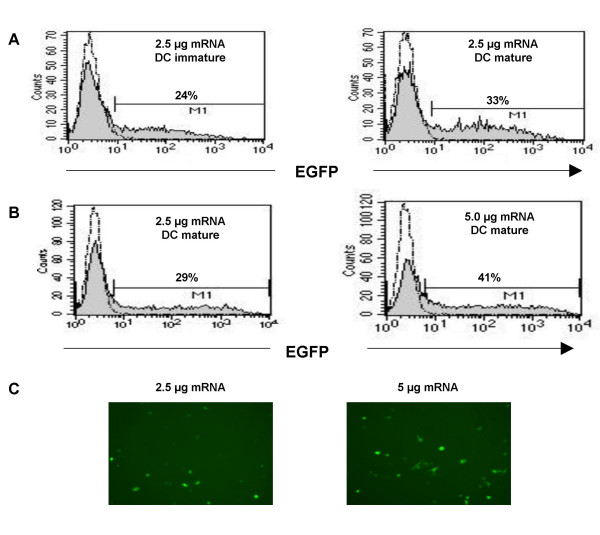
***Transfection of immature DCs with EGFP mRNA***. iDCs were transfected by lipofection using DMRIE-C and *in vitro*-transcribed EGFP mRNA. **A**) 2.5 μg EGFP mRNA/0.5 × 10^6 ^iDCs were transfected and the DCs were kept either as immature cells or matured using a cytokine cocktail. **B**) 2.5 μg and 5.0 μg EGFP mRNA/0.5 × 10^6 ^iDCs, respectively, were transfected and the DCs matured. EGFP expression was analyzed by flow cytometry 48 h after transfection (grey histograms). DCs transfected with H_2_O only were used as a control (broken line). **C) **EGFP-expression in transfected DCs was visualized using a fluorescent microscope.

Different techniques are discussed in the literature for loading DCs with RNA (Step 3) [30-36] but the optimal procedure for generating fully immunocompetent RNA-expressing DCs for *in vivo *application still has to be defined. In our studies, we utilized the method of lipofection to load DCs with tumor-derived RNA. To quantify the efficiency of RNA transfection, iDCs were lipofected with *in vitro*-transcribed EGFP mRNA using the transfection reagent DMRIE-C at day 7 of culture. DMRIE-C was chosen since it showed the lowest toxicity and highest transfection efficiency compared to other transfection reagents, such as DOTAP and TransFast (data not shown). Flow cytometric analysis of EGFP expression 48 h post-transfection revealed an average transfection rate of 20 – 40%, depending on the conditions chosen (Fig. 3).

In addressing the question whether subsequent maturation of transfected DCs had an impact on protein expression, we found an increase in EGFP-positive cells when transfected DCs (2.5 μg mRNA/0.5 × 10^6 ^cells) were exposed to the maturation cocktail for 48 h (Fig. 3A). The lipofection efficiency could also be enhanced by application of increasing amounts of EGFP mRNA. For example, the use of 5.0 μg mRNA/0.5 × 10^6 ^cells increased the percentage of positive cells by 10% compared to 2.5 μg mRNA (Fig. 3B). In all cases, lipofection of mRNA resulted in a broad range of protein density, generating DCs with EGFP protein expression ranging from low to very high levels (Fig. 3A, B and 3C).

### Lipofection of DCs with RNA does not adversely interfere with cell maturation

**Figure 4:**
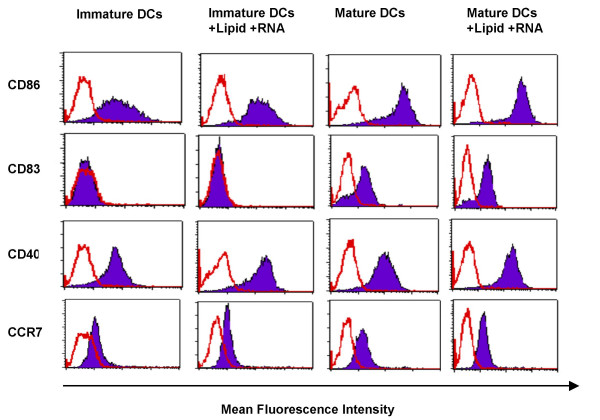
***Lipofection of DCs with RNA does not adversely affect their phenotype***. iDCs were incubated with DMRIE-C plus RNA, as described in the *Method*s section. After a 4 h incubation, the DCs were either kept immature or matured using a cytokine-cocktail. The DC phenotype was analyzed by flow cytometry 48 h after transfection.

Since we were interested to know whether RNA lipofection of iDCs interfered with DC maturation, we co-incubated the transfected iDCs with the different lipofection components (lipid + RNA), with or without maturation cytokine cocktail, and analyzed the DC phenotype by flow cytometry 48 h later. Incubation of iDCs with lipid plus RNA had no significant impact on DC maturation (Fig. 4, two left panels). A fully mature DC phenotype could only be induced by incubation with the pro-inflammatory cytokine cocktail, resulting in high expression of CD40 and up-regulation of CD86 and CD83 expression. Also, expression of the chemokine receptor CCR7, which is crucial for the migration of DCs to secondary lymphoid organs [37-40], was significantly up-regulated only after cytokine-induced maturation of the DCs (Fig. 4, two right panels).

Additionally, we analyzed the cytokines secreted by the DCs in order to determine whether loading of iDCs with RNA altered their cytokine production capacity. We tested supernatants of differentially treated DCs using a cytokine multiplex panel. On day 7 of culture, iDCs were incubated with the different components (lipid alone, RNA alone or RNA in combination with lipid). Subsequently, DCs were either matured or kept in the immature stage for 48 h before supernatants were harvested and analyzed. IL-12p70 was detected at low levels while IL-10 and IL-2 were detected in slightly higher amounts, but neither the applied RNA nor the lipofection reagent showed any significant impact on the levels of cytokines secreted by iDCs or mDCs. In general, maturation of DCs led to an increased secretion of these three cytokines (data not shown).

### Specific effector-memory CTLs can be activated by DCs lipofected with tumor cell line-derived RNA

DCs applied for vaccination (Fig. 1, Step 4) must be competent to stimulate specific immune responses. To evaluate the specific immunostimulatory potential of RNA-loaded DCs for effector-memory CTLs, iDCs derived from HLA-A*0201-positive healthy donors were transfected on day 7 with total RNA prepared from the tumor line RCC-26 using lipofection (5.0 μg RNA/0.5 × 10^6 ^DCs). Following transfection, DCs were matured for 48 h. On day 9, transfected DCs were co-cultivated with TIL-26 cells. TIL-26 line is a tumor antigen-specific CTL line that was expanded from the tumor-infiltrating lymphocyte population of patient-26 and was demonstrated to recognize a RCC-26-associated determinant presented by HLA-A*0201-encoded molecules [14]. The TIL-26-derived clone, TIL-26(GG) [41] was employed for all experiments. After a 24 h incubation period, the supernatants of TIL-26(GG) co-cultured with DCs were harvested and assessed for IFNγ in a standard ELISA assay.

**Figure 5:**
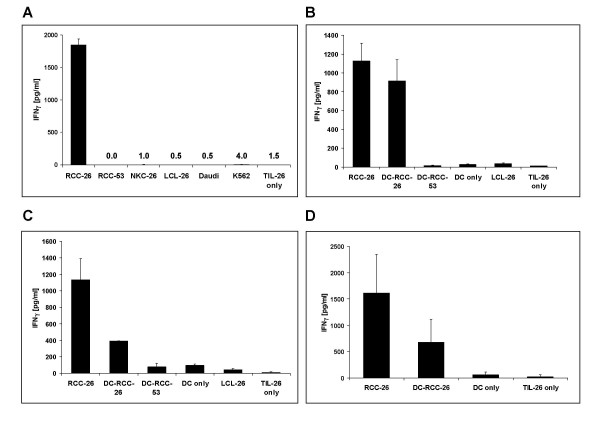
Reconstitution of the TIL-26 epitope in HLA-A*0201-positive DCs after lipofection with RCC-26-derived total RNA. **A) **To demonstrate the specificity of TIL-26(GG) for an epitope provided by RCC-26, different tumor cell lines were co-cultivated with the T cell clone in a ratio of 5:1 and supernatants were analyzed 24 h later. **B, C and D) **HLA-A*0201-positive iDCs were transfected using DMRIE-C with total RNA derived from RCC tumor cell lines. 48 h after transfection and maturation the mDCs were co-cultivated with the TIL-26(GG) T cell clone at a ratio of 5:1 for 24 h. B and C show two individual experiments contributing to the diagram in D, showing pooled data for four independent experiments. All experiments were carried out in triplicates and activation of T cells was measured by analysis of IFNγ secretion.

To demonstrate specificity, the TIL-26(GG) clone was co-cultivated with different cell lines. Only co-culture with RCC-26 cells, but not with autologous LCL-26, the normal kidney cell line (NKC-26), or the HLA-A*0201-matched tumor cell line RCC-53 induced IFNγ-release, indicating that only RCC-26 cells provided the specific ligand for TIL-26 activation (Figure 5A). Furthermore, TIL-26(GG) had no LAK or NK activitiy directed against Daudi or K562 cells. DCs transfected with the RCC-26-derived RNA, but not DCs alone, were able to induce IFNγ-secretion in TIL-26(GG) cells, indicating that the RCC-26-derived RNA was also capable of providing the TIL-26-specific epitope to the DCs. DCs transfected with RNA derived from RCC-53 or LCL-26, both of which are HLA-A2-positive cell lines that do not express the TIL-26 epitope (Fig. 5A), were not recognized by the T cell clone, further confirming the specificity of the T cell activation by RNA-pulsed DCs (Fig. 5B and 5C). RNA-loaded DCs prepared from HLA-A*0201-negative donors were also unable to specifically reactivate TIL-26 cells (data not shown).

Among individual experiments, responses stimulated by DCs reached levels ranging from 20% – 80% of those seen with stimulation by the RCC-26 cell line. These variations in the stimulation capacity of the RNA-loaded DCs might possibly be a consequence of variations in the donor DCs that were not readily apparent from characterization of the DC phenotype. Alternatively, differences in the prevalence of epitope-specific RNA in various RNA preparations or differences in transfection efficiencies may have contributed to these variations. In the optimal case, the TIL-26 activation capacity of RCC-26-transfected DCs was nearly as high as the stimulating potential of the RCC-26 cell line itself (Fig. 5B). Figure 5C shows another experiment in which RNA-loaded DC stimulation was only about 30% of the level of RCC-26 cells. These data are included in Figure 5D, providing pooled results of four independent experiments.

### Induction of RCC-26-specific CTL-lines using RNA-pulsed DCs

**Figure 6:**
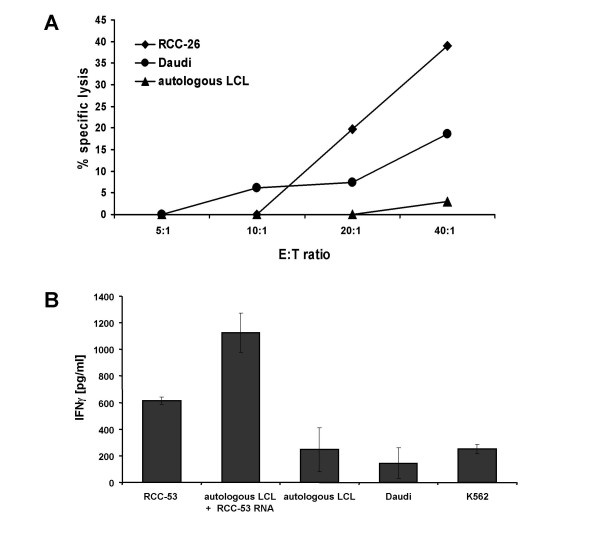
***Induction of RCC-specific CTLs using RNA-transfected DCs as APCs***. Monocyte-derived DCs were transfected with total RNA (5 μg/0.5 × 10^6 ^DCs) isolated either from RCC-26 (A) or RCC-53 (B) tumor cell lines. After a maturation period of 48 h the DCs were co-cultivated with autologous PBMCs (PBMC/DC ratio = 8:1) for 10–12 days following three rounds of restimulation (PBMC/DC ratio = 4:1). In the case of RCC-53 specific CTLs (B) an additional round of restimulation was carried out using autologous LCLs transfected with RCC-53-derived total RNA. The PBMC/DC co-culture was supplemented with IL-7 and IL-2 as described in the *Methods *section. **A) **RCC-26-specific CTLs were harvested three days after the last round of restimulation and specific lysis was analyzed in a standard 4-h ^51^Cr-release assay. **B) **RCC-53-specific T cells were harvested 10 days after the last round of restimulation and specificity of the T cells was determined by measuring IFNγ in the 24 h supernatant media T cell/target cell co-cultures (1.5 × 10^4 ^T cells : 1.5 × 10^4 ^target cells per 200 μl).

To analyze the capacity of RNA-pulsed DCs for *de novo *priming of antigen-specific T cell responses, we generated DCs from HLA-A*0201-positive healthy donors and used them as APCs after lipofection with total RNA derived from either the RCC-26 or RCC-53 cell lines (5.0 μg RNA/0.5 × 10^6 ^DCs). Autologous PBMCs were cocultivated with the transfected mDCs in a ratio of 8:1 during the first round of stimulation and in a ratio of 4:1 thereafter. We found that DCs transfected with either RCC-26- or RCC-53-derived RNA functioned as potent APCs and were able to induce RCC-26- and RCC-53-associated T cell responses, respectively, after three (in the case of RCC-26) or four (in the case of RCC53) rounds of weekly restimulation (Fig. 6).

The CTL line induced with RCC-26 RNA-transfected DCs was analyzed using a 4-h ^51^Cr-release assay. They showed an efficient specific lysis of the RCC-26 cell line itself but not of autologous unloaded LCL-26 cells (Fig. 6A). Likewise, the T cell line primed with RCC-53-loaded RNA was activated specifically to secrete IFNγ using either RCC-53 tumor cells or autologous LCLs pulsed with RCC-53-derived total RNA (Fig. 6B). Re-activation of specific T cells by RNA-transfected autologous LCLs thereby can be instrumental for analyzing specific T cell responses developing as a consequence of DC vaccination (Fig. 1, Step 6). In fact, it was possible to maintain the RCC-53-specific T cell line with autologous LCLs transfected with RCC-53-derived RNA. These observations indicate that continuous preparation of DCs from patient PBMCs either for analysis of T cell responses or to maintain T cell lines *ex vivo *may not be required.

In both examples, Daudi and/or K562 cells were used to measure non-MHCrestricted lysis or cytokine secretion, respectively. A low level of activity was detected in the uncloned T cell populations but this was decidedly lower than the specific reactivation achieved using the corresponding tumor cell lines.

## Discussion

In this report we present our strategy for the development of a generic RNA-pulsed DC vaccine for mRCC. Patients with mRCC have a poor prognosis and few treatment options; therefore, most clinical trials analyzing the efficacy of cell-based vaccines have studied the treatment of patients with advanced disease. A DC-based vaccine presenting RCC-associated antigens comprises a promising approach for generating antitumor responses, since DCs are the most potent antigen-presenting cells and they are capable of *de novo *priming of antigen-specific CD4 and CD8 effector cells from naïve T cells *in vivo *[reviewed in [42,43]]. Myeloid DCs can be generated from monocytes when cultured in the presence of GM-CSF and IL-4 *ex vivo *and thereby they are easily accessible in considerable numbers [44].

In contrast to melanoma where different peptide-specific vaccination approaches have been studied in numerous clinical trials, less is known about TAAs in RCC. Therefore, most clinical trials initiated to evaluate the safety and feasibility of DC-based vaccines for RCC so far have utilized DCs that were loaded with autologous tumor lysates [17,18,45-47]. This approach has the disadvantage that patient material must be available and often may be limited. As an alternative, two clinical trials have utilized tumor lysates prepared from established allogeneic RCC lines, which overcomes this handicap by providing a generic source of tumor cells for preparation of tumor lysates [18,48]. However, it is unclear how efficiently various TAAs are transferred by cell lysates. An attractive alternative is to utilize DCs transfected with defined or total tumor-derived RNA. By applying RNA, transfer of TAAs can be quantified using surrogate marker RNA species expressed by the tumor cell lines, which is not possible when cell lysates are used [36].

A recent phase I clinical trial with mRCC patients who received immunizations with DCs expressing autologous tumor-derived RNA demonstrated the feasibility and lack of toxicity of such vaccinations [19]. In this study, the authors employed iDCs that were incubated with naked RNA on the day of administration, relying on the natural capacity of DCs to take up RNA. Based on the finding that iDCs may induce tolerance [49,50], the use of mDCs would now seem to be a better strategy.

Several different methods for achieving RNA delivery to DCs have been analyzed to date, whereby electroporation and lipofection of DCs have been investigated most intensively [19,28,30-36]. The type of delivery that yields DCs capable of eliciting optimal T cell responses *in vivo *has yet to be determined. Our own studies revealed that electroporation yielded higher percentages DCs expressing protein, however, the levels of protein expression on a per cell basis were higher in lipofected DCs [36]. How these differences impact on *de novo *priming of T cells remains to be determined.

Here, we utilized DCs transfected with RCC-26-derived total RNA using the technique of lipofection. We selected the cationic lipid reagent DMRIE-C, since it provided the most efficient transfer of RNA and showed the lowest DC toxicity. We achieved a transfection rate of up to 40% when iDCs were transfected with EGFP mRNA and subsequently matured using a cytokine cocktail. It has been reported recently that comparatively high transfection efficiencies could also be obtained with other transfection reagents [34]. Here, the authors demonstrated an average transfection efficiency of 47%, with a viability of 92%, when mature DCs were transfected with an *in vitro*-transcribed EGFP RNA.

Some studies revealed that transfection with mRNA could partially activate DCs, presumably due to double-stranded secondary structures of the mRNA and toll-like receptor signalling [51-53]. Therefore, we were interested in the question whether the components used during our procedure of lipofection would influence the maturation status of the DCs. We found no evidence that application of lipid and RNA, at the concentrations used in our experiments, led to significant DC activation. Thereby, incubation with a defined pro-inflammatory cytokine cocktail was still necessary to induce a distinct mature DC phenotype. Furthermore, transfection of DCs with mRNA had no significant influence on the cytokines produced by the DCs. Thus, neither DC phenotype nor cytokine secretion levels were modulated by the lipofection components and/or procedure.

To achieve effective T cell responses in patients, DCs generated *ex vivo *should be competent both in re-activating pre-existing effector-memory CTLs and in priming specific T cells *de novo*. Antitumor CTL responses can often be detected in RCC patients [3-7,41]. However, these T cell responses are insufficient in disease control, possibly due to a late developing general immune suppression or due to the encounter of such T cells with defective antigen-presenting cells [54-60]. Stimulation of memory T cells with antigen-loaded DCs generated *ex vivo *may overcome these limitations and enhance tumor-specific T cell immunity in RCC patients.

To test whether DCs lipofected with RCC-derived RNA could reactivate specific CTLs, we used the tumor-specific T cell clone, TIL-26(GG), which is HLA-A2- restricted and specific for a TAA expressed by RCC-26 cells [14,41]. We could show that DCs lipofected with RCC-26-derived RNA were highly competent in reactivating these TIL-26 cells. In contrast, DCs transfected with RNA derived from RCC-53, a cell line which does not express this particular TIL-26 epitope, were unable to activate TIL-26(GG) cells, demonstrating the specificity of this response. We found that RNA-transfected DCs varied in their capacity to restimulate TIL-26 cells, often being weaker than RCC-26 tumor cells. We have shown elsewhere [36] that transferred RNA has a very short half-life in DCs so that peptides for MHC presentation will presumably only be available from one round of protein synthesis. Variations in ligand reconstitution on DCs thereby will be dependent upon the amount of RNA transferred to the DCs whereas tumor cells can generate new transcripts that can provide a continuous supply of peptides for MHC presentation.

A second important functional characteristic of RNA-pulsed DCs, namely their capacity to prime specific T cells *de novo*, was also demonstrated in our studies. We found that DCs lipofected with RCC-26- or RCC-53-derived RNAs were capable of inducing specific T cell responses against the corresponding tumor cell lines. Phenotype analysis of *de novo *primed T cell populations revealed that they contained both CD4^+ ^and CD8^+ ^T cells. Greater expansion of CD8^+ ^T cells occurred during restimulation, ultimately yielding altered CD4^+^/CD8^+ ^ratios when compared to naïve lymphocyte populations of healthy donors (data not shown).

Although, the *in vitro*-primed T cell lines showed some non-MHC-restricted activity directed against NK-sensitive K562 or LAK-sensitive Daudi cells, substantially stronger responses were detected against the specific tumor cell lines whose RNA was used for priming. In other studies, we demonstrated that non-MHC-restricted T cells and NK cells may play a significant role in anti-RCC immunity [61,62]. Therefore, we consider the induction of these innate effector cells by the DCs to be an advantage.

One critical aspect in generating and characterizing specific T cells is the need for high numbers of DCs for use as potent APCs. During the generation of the RCC-53-specific T cell line, autologous LCLs transfected with RCC-derived RNA could be successfully substituted as APCs for restimulation, in addition to being used for function and specificity studies. This demonstrated that RNA-transfected LCLs could efficiently provide pMHC ligands of interest and thereby provided an easily accessible alternative to RNA-loaded DCs. However, it must be cautioned that LCLs could replace DCs only after specific T cell responses had developed in order to avoid reactivation of EBV-specific T cells. Cultures can be established under limiting dilutions conditions in order to separate T cells with different specificities. Alternatively, the use of mini-EBV constructs that encode a limited number of EBV proteins, yet still retain the capacity to immortalize B cells, provides an elegant means to reduce this handicap [63].

A more decisive point in analyzing responses to a generic vaccine based on total tumor RNA is that autologous LCLs can provide a helpful tool for elucidating the fine specificity of T cell responses. Following loading with RNA, they can present epitopes in both class I and class II molecules. Furthermore, LCLs can be transfected with any TAA mRNA of interest in order to assess the emergence of TAA-specific T cells following vaccination. Previous clinical trials have indicated that following DC vaccination T cell responses could be detected against oncofetal antigen [18,19], human telomerase reverse transcriptase (hTERT) [19] and G250/CA-IX antigens [19]. Transfection of LCLs with mRNA encoding these specific TAAs could be useful for evaluating specific T cell responses.

Two GMP-certified, genetically engineered RCC-26 cell lines, RCC-26/B7.1/IL-2 and RCC-26/B7.1/IL-7, are currently studied in phase I clinical trials [13,23]. These tumor cell lines could easily serve as sources of GMP-quality RNA for further development of the proposed generic DC-based vaccine approach. Findings concerning feasibility, toxicity and potential induction of autoimmunity emerging from these ongoing phase I trials will also be useful for designing a clinical trial based on RNA-loaded DCs. RNA generated from these GMP-certified RCC-26 cell lines will encode an array of TAAs present in the tumor cells. For example, both vaccine variants have been demonstrated to show over-expression of PRAME, oncofetal antigen, adipophilin and hTERT RNAs [23], all of which have been often found to be over-expressed in fresh tumors and to serve as targets for T cell responses [11,18-21,64,65]. The immune monitoring tools specific for such molecules that prove useful for defining T cell responses that develop following tumor cell vaccination also can be used for analyzing specific T cell responses to the generic DC-based vaccine proposed here. As more information emerges regarding specific TAAs that can be used to target immune responses to RCC, it will be possible to develop defined vaccines based on the use of pools of RNA encoding selected TAAs. Such an approach has recently been described for melanoma [35].

## Conclusion

We describe a DC-based vaccine strategy for RCC patients using a generic source of RNA derived from a well characterized tumor cell line. When DCs derived from HLA-A2-positive donors were loaded with tumor cell line-derived RNA using the method of lipofection, they were able to re-activate effector-memory CTLs and were also capable of priming T cells *de novo *to mediate cytotoxicity or secrete cytokine after contact with RCC cells. The availability of GMP-certified cells, expressing a variety of TAAs that are shared by many RCC and can provide target epitopes for T cells will allow this strategy to be more easily adapted for clinical testing. Furthermore, the ability to load tumor cell line-derived RNA into autologous LCLs provides a useful tool for immune monitoring.

## Competing interests

The author(s) declare that they have no competing interests.

## Authors' contributions

CG and SR developed the RNA-loaded DCs and performed the functional studies, they analyzed the data and drafted the manuscript.

AW carried out the experiments using RNA-loaded DCs for *de novo *priming of T cells.

EN provided the TIL-26(GG) cells for assessment of effector-memory T cell responses.

DJS established the RCC and TIL lines, conceived the vaccine strategy, participated in the design and analysis of the experiments and helped to draft the manuscript.

All authors read and approved the final manuscript.
